# Controlled ovarian stimulation in cancer patients under 18 years old; a case series

**DOI:** 10.1186/s13048-024-01352-0

**Published:** 2024-02-05

**Authors:** Azar Yahyaei, Mahdieh Moridi, Firouzeh Ghaffari

**Affiliations:** Department of Endocrinology and Female Infertility, Reproductive Biomedicine Research Center, Royan Institute for Reproductive Biomedicine, ACECR, Number 12, East Hafez Avenue, Banihashem Street, Resalat Highway, Tehran, Iran

**Keywords:** Fertility preservation, Oocyte cryopreservation, Controlled ovarian stimulation, Cancer patients, Adolescent pubescent girls, Case series

## Abstract

**Background:**

Fertility preservation for adolescent pubescent girls is a concern of the healthcare system and parents. Oocyte cryopreservation is regarded as a standard medical intervention for patients with a minimum age of 18 years. Evidence suggests that mature oocyte cryopreservation is possible for adolescent pubescent girls, although, ovarian stimulation for these patients remains a challenge.

**Cases presentation:**

This case series is the first report regarding ovarian stimulation with oocyte cryopreservation in younger than 18 years cancerous girls, who refer to ROYAN institute, Tehran, Iran, prior to the start of the treatment of cancer (November 2015 to February 2021). The oocyte cryopreservation was carried out in the 7 patients (five patients with Hodgkin lymphoma, one patient with Ewing sarcoma, and one patient with osteogenic tumor), the embryo cryopreservation in one patient with dysgerminoma, and the oocyte and embryo cryopreservation in one patient with germ cell tumor. No oocytes were retrieved after ovarian stimulation in the patient with medulloblastoma. For one of the patients with Hodgkin lymphoma, half of the tissues of one ovary were cryopreserved prior to ovarian stimulation.

**Conclusions:**

Oocyte cryopreservation is a feasible option of fertility preservation in the adolescent’s patients with cancer. However, only if reported acceptable fertilization rates, as well as the successful cases of live birth from oocyte cryopreservation at the ages under 18, this option of preserving fertility can be applied to this age range.

## Background

Although the survival of children and adolescents with cancer has significantly increased in recent decades, cancer survivors suffer from long-term complications of this disease or its therapies, such as gonadotoxicity of therapies. The importance of enhancing this patient’s quality of life and preserving their ability to have a biological child in the future life highlights the necessity of fertility preservation (FP) strategies. Preserving the fertility of adolescent pubescent girls is a hot topic of discussion [1, 2].

The main options for FP in cancer patients include mature oocyte, or embryo, and ovarian tissue cryopreservation (OTC). Mature oocyte or embryo cryopreservation (EC) is a standard FP method in post-pubescent women if they have had adequate time for ovarian stimulation (OS). OTC is a FP method for pre-pubescent girls and post-pubescent patients who cannot delay the onset of cancer treatment [3, 4]. Previously, it was considered experimental; however, the American Society for Reproductive Medicine (ASRM) proposed OTC as an established method [5]. Although pediatric research must continue [6]. This technique is more invasive than other FP methods as it needs laparoscopy and, if necessary, laparotomy to remove and re-graft the ovarian tissue; there is also the risk of tissue contamination with cancer cells and the transmission of the disease following tissue graft [3, 4].

Recent advances in the cryobiology significantly promoted oocyte cryopreservation (OC), while lack of sperm dependence has made this method a desirable option. OC is regarded as a standard medical intervention for patients with a minimum age of 18 years, although several studies introduced it enough safe to use for younger patients [7–17].

Although evidence suggests that mature OC is possible for adolescent pubescent girls, it is controversial due to the dearth of research on OS in these patients. The controversial topics related to OS and mature OC in the cancer affected adolescents that included the unknown success rate of cryopreserved oocyte usage in this age group [7]; no experience of sexual intercourse, which may cause psychological problems for them [17]; parent decision-making on behalf of the patient; inadequate knowledge about adolescents’ response to OS; adolescents finding it difficult to accept this process due to the necessity of daily injections, ultrasound, and a series of lab tests [18]; the possible immaturity of the hypothalamus-pituitary-ovarian (HPO) axis that may lead to an inappropriate ovarian response; the unreliability of factors that determine the prescribed gonadotropin dose for OS in this age group, including anti-mullerian hormone (AMH) and antral follicle count (AFC) [10, 19]; and inherent differences in the adolescent ovary physiology, especially in the first post-pubescent years [17].

The present study reports cases of OS in cancer patients younger than 18 years in ROYAN institute, Tehran, Iran (2015-2021). As well as, reporting the outcomes of OS cycles for OC, the associated challenges in this age group are also discussed.

## Cases presentation

**Fig. 1:**
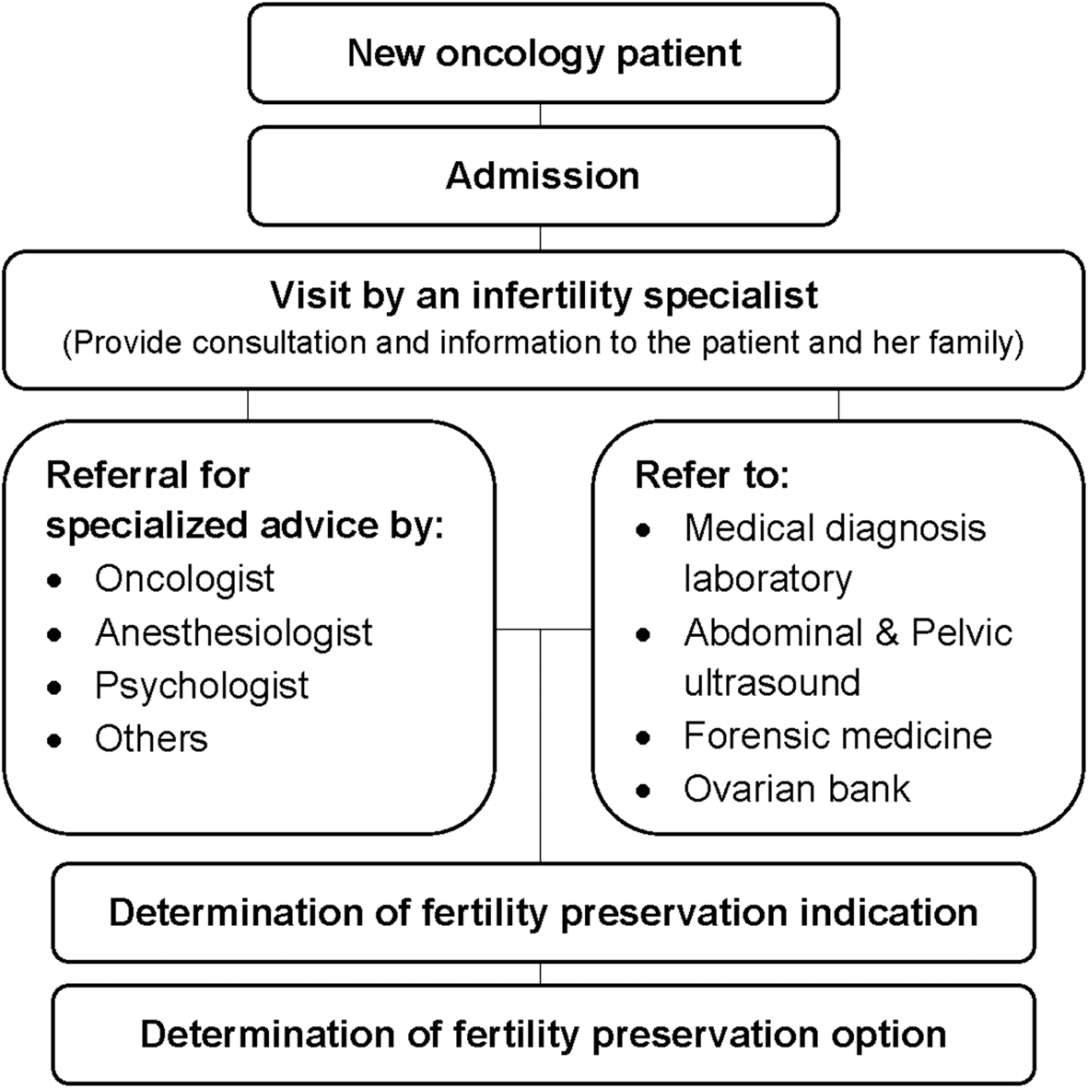
The process of admission oncofertility cases in ROYAN Institute

This case series study was approved by the Research Ethics Committee of ROYAN institute, Tehran, Iran (Ethics Approval Code: IR.ACECR.ROYAN.REC.1400.080). Between November 2015 and February 2021, the patients of our Oncofertility and FP Clinic, Royan institute, Tehran, Iran, who have the inclusion criteria, were invited in this study. Our main criteria were included (1): younger than 18 years (2): refer before cancer therapies. The process of admission oncofertility cases in this center is presented in Fig. 1.

Ten patients between the ages of 14 and 17, and 1 to 6 years from their Menarche age (the first menstrual cycle) entered the cycle of the OS. These patients suffered from Hodgkin Lymphoma (HL) (*n*=5), ovarian tumor (germ cell tumor and dysgerminoma) (*n*=2), Ewing sarcoma (*n*=1), osteogenic tumor (*n*=1) and brain tumor (medulloblastoma) (*n*=1). The OS was performed for all 10 patients using the standard gonadotropin releasing hormone (GnRH) antagonist protocol. The OS beginning was different among patients: in the early proliferative phase (*n*=8), during the late proliferative phase (*n*=1), and in the luteal phase (*n*=1).

Trans-abdominal ultrasound was used successfully for follicular monitoring. The gonadotropin prescribed for all patients was recombinant follicle stimulating hormone (r-FSH) (Gonal-f) (Follitropin alfa, Merck Serono, Germany): for eight patients began with a dose of 150 IU/day, for the patient in the luteal phase with a dose of 225 IU/day, and for the patient with medulloblastoma with a dose of 112.5 IU/day. The prescribed drug for triggering final oocyte maturation was recombinant human chorionic gonadotropin (r-HCG) (500 µg Ovitrelle) (Choriogonadotropin alfa, Merck Serono, Switzerland) in the patients who underwent OS during 2015-2018 and low-dose GnRH-agonist (0.2 mg triptorelin) (Decapeptyl, Ferring Pharmaceuticals, Germany) for the other patients.

The OC was carried out in 7 patients, the EC in one patient and the oocyte and embryo cryopreservation in one patient. No oocytes were retrieved after OS in the patient with medulloblastoma. For one of the patients with HL, half of the tissues of one ovary were cryopreserved prior to OS. The two patients with ovarian tumor had a history of unilateral oophorectomy.

Baseline characteristics of the patients are presented in Table 1. Information of patients' OS cycle and its results is presented in Table 2. As well as, the summary of similar studies is presented in Table 3.

**Table 1:** Baseline characteristics of the patients

**Case**	1	2	3	4	5	6	7	8	9	10
**Year**	2015	2017		2018		2019			2020	2021
**Diagnosis**	HL^c^	Ovarian tumor (Germ cell)	HL	HL	Ewing Sarcoma	Ovarian tumor (Dysgerminoma)	HL	Brain tumor (Medulloblastoma)	Osteogenic tumor	HL
**Age** (year)	16	16	17	17	16	15	17	16	14	17
**Menarche age**	12	13	N/A	11	10	13	14	12	13	11
**BMI** (kg/m^2^)	19.1	19.9	18.1	20.3	19.2	19.5	19.6	19.1	24.9	19.1
**Ovarian surgery**^a^	Cryopreservation of ½ left ovarian tissue^d^	Right Oophorectomy	No	No	No	Right Oophorectomy	No	No	No	No
**Radiotherapy**^b^	No	No	No	No	No	No	No	No	No	No
**Chemotherapy**^b^	No	No	No	No	No	No	No	No	No	No
**Menstruation**	Regular	Irregular	Irregular	Irregular	Regular	Irregular	Regular	Irregular	Irregular	Regular
**AFC**	4	6	16	14	16	6	14	20	20	20
**AMH** (ng/ml)	N/A^e^	0.4	0.9	0.9	5.1	1.9	N/A	8.9	N/A	6.0
**FSH** (IU/L)	3.7	4.2	9.1	9.1	3.4	6.7	4.5	3.9	9.0	2.2
**LH** (IU/L)	9.4	8.8	7.8	5.3	2.6	12.4	3.2	0.6	12.5	5.0
**Prog** (ng/ml)	0.2	2.5	0.2	1.1	N/A	0.5	N/A	0.2	0.2	1.2
**E2** (pg/ml)	88.3	141.9	40.3	188.0	N/A	33.2	N/A	17.5	144	N/A

^a^History of Ovarian surgery before ovarian stimulation

^b^Radiotherapy and/ or chemotherapy history before ovarian stimulation

^c^Hodgkin lymphoma

^d^Cryopreservation of ovarian tissue with the aim of fertility preservation

^e^Not Available

**Table 2:** Information of patients' ovarian stimulation cycle and its results

**Case**	1	2	3	4	5	6	7	8	9	10
**COS Protocol**	GnRH antagonist									
**Stimulation cycle start**	Early. P^b^	Luteal Phase^c^	Early. P^d^	Late. P^e^	Early. P					
**Gonadotropins**	recombinant-FSH									
**Starting gonadotropin dose (IU)**	150	225	150	150	150	150	150	112.5	150	150
**Total gonadotropin dose** (IU)	1050	2025	1350	1500	1200	1650	1425	1687.5	1350	1350
**GnRH-antagonist** (ampule)	4	4	5	6	4	8	3	9	6	5
**Duration of stimulation** (day)	8	10	10	11	9	12	9	14	10	11
**Follicle> 12 mm**^a^	10	6	12	11	13	17	11	13	8	22
**Triggering medication**	2recombinant-HCG					Low dose GnRH-agonist				
**Total oocytes retrieved**	13	10	9	10	21	6	8	0	32	33
**Total mature oocytes** (MII)	13	10	9	8	17	5	5	0	26	23
**Total oocytes cryopreservation**	13	5	9	8	20^f^	0	7^f^	0	26	23
**Total embryos cryopreservation**	-	5	-	-	-	5	-	-	-	-
**OHSS & others side effects**	No									

^a^In the day of triggering final oocyte maturation

^b^Proliferative

^c^Progesterone >1.5 ng/ml

^d^Larges follicle <13 mm & progesterone <1.5 ng/ml

^e^Larges follicle >13 mm & progesterone <1.5 ng/ml

^f^MII+GV (germinal vesicle)

**Table 3:** The summary of similar studies

**Author; Year**	Kutteh et al, 2018 [9]			Peddie et al, 2018 [16]	Lavery et al, 2016 [10]								Oktay et al, 2014 [14]					Reichman et al, 2012 [17]	Kim et al, 2011 [8]	Oktay et al, 2010 [15]		Noyes et al, 2009 [13]	Nagashima et al, 2005 [12]	
**Patients**	3	2	1	1	8	7	6	5	4	3	2	1	5	4	3	2	1	1	1	1		1	2	1
**Diagnosis**	Medulloblastoma			myelodysplastic clone^f^	Sickle cell anemia								ALL	Germ cell tumor	Turner syndrome			Myelodysplastic syndrome	SPH caused by TGV^d^	Turner syndrome		Ovarian luteinized the coma	AML	17
**Age (Menarche age)**	14 (13)	16 (12)	14 (13)	14 (12)	18	18	17	16	16	16	15	14	14 (13)	15 (13)	14 (11)	13 (12)	13 (13)	13	17	14 (11)		19	18	No
**Ovarian surgery**	No	No	No	No	No	No	No	No	No	No	No	No	No	Left oophorectomy	No	No	No	No	No	No		Left oophorectomy	No	No
**Radiotherapy**	No	No	No	No	No	No	No	No	No	No	No	No	No	No	No	No	No	No	No	No		No	No	Yes
**Chemotherapy**	No	No	No	No	No	No	No	No	No	No	No	No	Yes	No	No	No	No	No	No	No		No	No	N/A^a^
**AMH** (ng/mL)	12.5^g^	6.8^g^	13.3^g^	N/A	16^e^	N/A	24^e^	23.8^e^	N/A	N/A	N/A	N/A	0.8	1.6	0.9	0.76	1.59	0.95	N/A	0.9		N/A	N/A	N/A
**FSH** (mIU/mL)	N/A	N/A	N/A	7.1	2.9	7.6	4.2	7.6	4.3	1.2	4.8	2	7.8	5.6	5.3	5.6	5.7	5.0	N/A	5.3		N/A	N/A	N/A
**LH** (mIU/mL)	N/A	N/A	N/A	10.4	3.2	3.9	3.6	3.8	3.3	0.8	6.2	6.2	8.1	9.2	9.5	5.3	3.9	2.9	N/A	9.5		N/A	N/A	N/A
**AFC**	31	15	25	17	12	20	20	16	16	18	6	13	5	11	12	6	6	9	N/A	12		N/A	N/A	N/A
**COS Protocol**	GnRH-antagonist			GnRH-antagonist							GnRH-agonist		GnRH-antagonist					GnRH-antagonist	GnRH-agonist	GnRH-antagonist		GnRH-agonist	N/A	N/A
**Duration of stimulation**	10	12	11	N/A	12	10	11	10	10	11	10	14	12	11	10	10	11	N/A	N/A	N/A		N/A	N/A	N/A
**Gonadotropins**	HMG			HMG	r-FSH								r-FSH (+HMG or r-LH)					HMG	r-FSH (+HMG)	r-FSH (+HMG)		N/A	N/A	N/A
**Starting gonadotropin dose (IU)**	150	225	150	225	225	187.5	150	150	112.5	112.5	150	150	N/A	N/A	N/A	N/A	N/A	225	N/A	225 (+150)^c^	225 (+150)^b^	N/A	N/A	N/A
**Total gonadotropin dose** (IU)	1800	1800	1950	N/A	3075	1875	3350	1500	1462.5	1312.5	1875	2625	1775	2137.5	2075	2250	2625	N/A	N/A	N/A	N/A	N/A	N/A	N/A
**Triggering medication**	GnRH-agonist			r-HCG	r-HCG				GnRH-agonist		r-HCG		GnRH-agonist			r-HCG	u-HCG	r-HCG	N/A	GnRH-agonist		N/A	N/A	N/A
**Total oocytes retrieved**	26	18	25	13	7	31	5	14	29	21	5	7	21	2	11	16	19	20	14	7	11	38	0	0
**Total mature oocytes** (MII)	17	12	23	12	1	30	3	11	25	16	4	7	10	4	8	7	9	8	8	4	8	N/A	0	0
**OHSS**	No	No	No	N/A	No	Yes	No	No	No	No	No		N/A	N/A	N/A	N/A	N/A	N/A	N/A	N/A		N/A	N/A	N/A

^a^Not Available

^b^The first cycle

^c^Second cycle

^d^Secondary pulmonary hypertension caused by transposition of great vessels

^e^pmol/L

^f^Myelodysplastic/pre-malignant clone with monosomy 7

^g^nmol/L

## Discussion and conclusions

According to American Society of Clinical Oncology (ASCO) guideline all newly diagnosed cancer patients of reproductive age should be informed about potential loss of fertility and should refer to infertility specialists [20]. Recommendation of gamete and/or embryo cryopreservation before gonadotoxic treatments is currently a standard of care [21]. FP in the adolescent patients is very rare and typically performed before gonadotoxic therapies for oncologic or non-oncologic conditions, such as a stem cell transplantation, or impending premature ovarian failure (POF) [7].

There are many dilemmas about FP among adolescent populations, including parental decision-making on behalf of the patient, adolescent decision capacity [22], and loss of virginity due to the trans-vaginal approach during oocyte retrieval. The latest one, loss of virginity is a too difficult condition for the parents, particularly in some cultural perspectives [10].

According to the instruction followed in our center, talking to adolescents about FP, including its necessity and steps, is done by a fertility specialist in the presence of their parents. The specialist explains these phases' step by step in a language understandable to them. Their questions are answered. If the adolescent and her parents’ consent, the parents are asked to sign the consent forms. Given the importance of virginity in Iranian Culture, our center adheres to the following instruction to prevent injuring the hymen during follicle puncture. After applying anesthesia the patient put in the lithotomy position, the infertility specialist separates the labia minora, making the hymen more visualized. Lubricates her little finger, and gently touches the edges of the hymen. And tries to dilate hymen when the little finger entered the vagina. Infertility specialist attempts to enter the ring finger and continue to dilate hymen and when the ring finger freely moves the physician tries middle finger and after that index and then thump and finally both index and middle fingers tries simultaneously. The procedure takes 10-15 minutes to perform. Now, a suitable speculum can be used without injuring the hymen. To eliminate the lubricant effects, the vaginal environment is washed with vigorous amount of normal saline and dried with a small sterile gauze before the insertion of the ultrasound probe. The remaining steps of follicle puncture will be similar to adults' instructions. In this study out of patients were virgin. And all of the intact hymen were annular shape. And none of them were injured during follicular puncture.

Knowledge gaps exist as to whether adolescence patients respond similarly to the OS or have similar outcomes, such as mature oocyte yield. Currently, published data in adolescents’ are limited to case series [10, 14, 23, 24]. Previous studies in the FP for oncology patients have shown lower oocyte yield in adult patients [25, 26]. Studies indicated the cancer pathology might itself induce ovarian reserve depletion [25]. As well as, cancer can induce an increased catabolic condition, increase in stress hormones with associated hypothalamic dysfunction; can predispose patients to lower levels of gonadotropins [18]. There are few studies that detail response to the OS and some of the studies had raised concern that adolescent patients might have a lower response to gonadotropins [7].

Although, sensitive markers of ovarian reserve, including AFC and AMH in estimating the dose of gonadotropins likely to yield maximum oocyte have been well documented, there is limited data of dose specific evaluation in pubertal girls [27]. As well as, there are arguments regarding the clinical value of AMH assessment in adolescent girls [28]. In our study, the correlation between AFC, AMH, gonadotropin dose, and the number of oocytes retrieved was not statistically significant, which could be partially attributed to the small sample size. Accordingly, judging the value of AFC and AMH for determining the gonadotropin dose and number of oocytes retrieved in the adolescent population requires a study with a larger sample size. Antral follicles are present in the ovaries of girls of all ages. At the onset of puberty, a rise in the FSH levels happens and antral follicles start growing. Primary menstruation cycles are frequently anovulatory, with regular ovulation occur later. While maturity of the hypothalamus-pituitary-axis is crucial for puberty, changes in ovarian maturity may also contribute. This may be reflected with little but constant transient reduction in the AMH level during puberty. There are significant numbers of follicles with abnormal morphology during puberty, these seem to be lost during adolescence. Follicle growth may also be different in their ovaries. Also, isolated secondary follicles from adolescents grew slower than those of adults that indicates an inherent maturity change. Although, its basis has remained unknown. Moreover, differences in local regulatory factors, that reflects the high density of small follicles in these young patients, may also contribute to the pattern of ovarian development. A discrepancy has been found among AFC, AMH and number of oocytes cryopreserved in some cases. These differences are likely multifactorial and could be a reflection of the ovarian development stage at extremes of youth or attributed to the effect of the chronic illness [10].

Although studies have suggested that trans-abdominal ultrasound may decrease the accuracy of AFC, overall this did not pose a significant issue in the adolescent treatment and had previously been successfully used for monitoring in the similar populations [10, 15–17]. In our study, the condition of adolescents' ovaries, including AFC, was examined by a skilled radiologist through the abdomen before the onset of the OS.

There are few studies in the adolescent patients that detail complications, delay in the initiating cancer treatment, and pregnancies after the completion of treatment. Most patients with cancer diagnosis can be afforded the 8-12 days required for the OS before they begin their treatment, without imperiling their oncologic care [17]. In our study, the OS delayed the adolescent cancer treatment by 10-14 days. It is worth noting that the cancer treatment delay was done after receiving confirmation from the oncologist. No complication was reported as a result of delayed cancer treatment.

The GnRH-antagonist protocol has decreased the interval between patient presenting and OS. The shorter duration of treatment, followed by a minimally invasive trans-vaginal oocyte retrieval, with a short recovery period, compared with laparoscopy, makes the procedure more acceptable to patients [10]. The use of the GnRH-antagonist protocol also provides the chance to utilize a GnRH-agonist for final oocyte maturation. Gonadotropin administration can begin at any point in the menstrual cycle. Response to medication will be evaluated with ultrasound (trans-vaginal or trans-abdominal, depending on the patient’s comfort level) and E2 measurements, with gonadotropin dosage adjusted accordingly. For an OS protocol, once the leading follicle grew to at least 12 mm in diameter or E2 reached 300 pg/mL, the patient began a daily injection of GnRH-antagonist to prevent ovulation [11].

It is anticipated that many females with age under 18 years may have an immature HPO-axis [11]. There is no decisive data, whether FSH alone or FSH plus luteinizing hormone (LH) should be used in these cases. Due to the predicted immaturity of the HPO-axis, FSH plus LH administration is the preferred approach [16]. Also, one option to limit the risk of ovarian hyperstimulation syndrome (OHSS) is considered a GnRH-agonist trigger instead of HCG. However, there are some concerns to use GnRH-agonists in the adolescent population because of the HPO-axis immaturity [17]. Therefore, it is imperative to check serum progesterone and LH levels ~ 8–14 h following a GnRH-agonist trigger to ensure that the triggering was effective [29, 30]. Typically an inadequate response is defined as a progesterone <3 ng/ml and an LH <15 IU/L [29].

Given the evidence so far equating a pregnancy rate of 6% per vitrified oocyte [31], around 20 oocytes being used per live birth [32]. The lower oocyte yields some oncologic patients deprived them of a good chance of pregnancy and live birth. However, in the poor response patients, there is the option of performing oocyte banking over a number of cycles [33]. Although, increasing the overall number of vitrified oocytes double stimulation during the follicular and luteal phases in the same cycle has been described successfully in these patients [34, 35].

Most studies on the OS outcomes and OC for adolescent girls with oncologic or non-oncologic diseases are limited to case reports [8, 13, 15–17] or case series [9, 10, 12, 14]. Based on the results of these studies, the hormonal profiles (FSH, LH, and AMH) and AFC of patients do not show significant differences in the adult patients. The OS protocol for patients have been OS with GnRH agonist or antagonist, which did not show a significant difference with adult patients in terms of duration of stimulation, gonadotropins dose, triggering medication, total oocytes retrieved, and total mature oocytes (MII).

Only Nagashima et al. reported no access to the oocyte in two patients (17 and 18 year old) with acute lymphoblastic leukemia (ALL) and acute myeloid leukemia (AML) [12]. Complications, such as OHSS, are very rare. Only Lavery et al. reported a case of OHSS that needed treatment support [10]. No OHSS case was observed in our study.

Kim et al. reported a live birth from the frozen oocytes retrieved from a 17-year old adolescent with secondary pulmonary hypertension caused by transposition of great vessels. After 5 years, 11 oocytes were fertilized under microinjection process. Two 5-day embryos were transferred to the patient. The outcome was the birth of a healthy boy at 38 weeks of pregnancy [8].

Manuel et al. carried out the OS and OC on the 41 oncologic patients between the ages 13 and 21 years old. Among them, 38 patients underwent a successful procedure of retrieving and freezing mature oocytes.

When dividing patients to 13-17 yr. group and 18-21 yr. group, there was no statistical difference in the AMH level, peak E2, gonadotropin dosage, duration of stimulation, total oocytes retrieved, mature oocytes retrieve, and cryopreservation between these two groups [11]. Hipp et al. compared the results of OS and OC in the patients under the age of 20 who were candidates for FP prior to gonadotoxic treatment with patients between the ages 20 and 29 years old. According to their report, even though the possibility of canceling the cycle of the OS due to the poor ovarian response is quite higher in the age group under 20, other factors of OS such as gonadotropins dosage, duration of OS, the number of retrieved oocytes, number of frozen mature oocytes are similar in both age groups [7].

In our study, the OS was performed in the five patients with HL. Comparison of these patients in terms of number of retrieved oocytes and mature oocytes suggests that the method of triggering final oocyte maturation is an effective factor in the number of mature oocytes.

In the present study, two patients had ovarian tumor (germ cell tumor and dysgerminoma) and a history of unilateral oophorectomy. Our oncologist consultation confirms the OS and vaginal oocyte retrieval in these patients. FP in ovarian cancer stands more challenging. In ovarian cancer patients, trans-vaginal oocyte retrieval carries a risk of ovarian capsule rupture and cancer cell spillage, which can cause staging up from 1A to 1C [36]. Bilateral disease is uncommon. The majority of patients have stage I disease, and nearly 90-95% of the cases are curable via post-operative chemotherapy) [37]. For the patient with germ cell tumor, the OS was carried out in the luteal phase and for the patient with dysgerminoma in the follicular phase. Ten oocytes were retrieved in the patient with germ cell tumor and 5 oocytes in the patient with dysgerminoma. Noyes et al. reported 38 retrieved oocytes in a 19-year-old patient with ovarian luteinized thecoma after OS with GnRH-agonist long protocol. Oktay et al. reported 8 retrieved oocytes (4 MII) in a 15-year-old patient with germ cell tumor after OS with GnRH-antagonist protocol. Contrary to our study, they used human menopausal gonadotropin (HMG) and/or r-LH in addition to r-FSH for OS, and utilized GnRH-agonist for triggering final oocyte maturation. In our study, use of different triggering methods could be the reason for retrieving different numbers of oocytes from the two patients.

In this study, no oocytes were collected after OS of medulloblastoma patient. While, Kutteh et al. reported OS performance for three medulloblastoma patients and, a retrieved desirable number of oocytes. They used HMG for the OS, whereas r-FSH was utilized in our research. Considering the effects of a brain tumor on the hypothalamic-pituitary axis, it seems that combined the use of r-FSH and HMG/r-LH, and also use of HCG instead of GnRH-agonist for triggering, could lead to retrieval of oocyte in this patient. Considering the possibility of incomplete maturity of the HPO-axis in adolescents, and its compensation by simultaneous administration of gonadotropins containing FSH and LH, and also more focusing on the triggering method including use of a “dual triggering” method, can be accompanied by retrieval of a larger number of mature oocytes.

Some studies have proposed that the approach of OTC simultaneously with oocyte or embryo cryopreservation be used as a solution for increasing the efficiency of FP methods. For this aim, laparoscopic surgery is performed to remove half of the tissues of one ovary and OS begins after one to two days. Research has shown that this method is not accompanied so far by any known complications and the number of collected oocytes does not decrease considerably after removal of the ovarian tissues [38, 39]. Here, we used the same method for a patient who suffered of HL and also, 13 mature oocytes retrieved.

In our center, FP has not been done for adolescents who are FP candidates for non-cancer indication such as those with thalassemia major, sickle cell anemia, rheumatoid diseases, and Turner syndrome. As a result, the present study did not compare the OS results of these cancer adolescents with non-cancer peers.

Based on our result, we suggested that, this option of FP is a feasible procedure for this age group. Among the available studies, only Kim et al. reported the success on OC for an adolescent patient. Other studies, and present research, provided the report regarding the information about the OS cycle, such as prescribed gonadotropins dosage, OS duration, probable side effects of the OS, the number of retrieved and frozen matures oocytes, and EC in some cases.

The results of the available studies resolved the issues regarding the subjects such as lack of sufficient knowledge regarding the adolescent patients’ response to OS, the possibility of failure of the HPO-axis to reach full maturity that might result in immature oocytes, plus, the uncertainty of the predictive factor of ovarian response in this age group including AMH and AFC. However, subjects such as uncertainty regarding the success rate of using the frozen oocyte from patients under the age of 18-year -old remain unanswered.

Based on the findings of the present study it is concluded that, OC is a feasible option in the adolescents. However, only if reported acceptable fertilization rates, as well as the successful cases of live birth from OC at the ages under 18, this option of preserving fertility can be applied to this age range. Therefore, we shall wait for more reports in the future regarding the use of frozen oocytes retrieved from patients under the age of 18-year-old, and then make the final judgment concerning the efficiency of this option for preserving fertility in this age range.

## Data Availability

All data generated or analyzed during this study are included in this published article [and its supplementary information files].

## Abbreviations

FP: Fertility preservation
OTC: Ovarian tissue cryopreservation
EC: Embryo cryopreservation
OS: Ovarian stimulation
ASRM: American Society for Reproductive Medicine
OC: Oocyte cryopreservation
HPO: Hypothalamus-pituitary-ovarian
AMH: Anti-mullerian hormone
AFC: Antral follicle count
HL: Hodgkin Lymphoma
GnRH: Gonadotropin releasing hormone
r-FSH: Recombinant follicle stimulating hormone
r-HCG: Recombinant human chorionic gonadotropin
ASCO: American Society of Clinical Oncology
POF: Premature ovarian failure
LH: Luteinizing hormone
OHSS: Ovarian hyperstimulation syndrome
MII: Mature oocytes
ALL: Acute lymphoblastic leukemia
AML: Acute myeloid leukemia
HMG: Human menopausal gonadotropin
